# Variation in physicochemical properties and bioactivities of *Morinda citrifolia* L. (Noni) polysaccharides at different stages of maturity

**DOI:** 10.3389/fnut.2022.1094906

**Published:** 2023-01-04

**Authors:** Jinlin Cai, Zijian Liang, Jian Li, Muhammad Faisal Manzoor, Hongsheng Liu, Zhong Han, Xinan Zeng

**Affiliations:** ^1^School of Food Sciences and Engineering, South China University of Technology, Guangzhou, China; ^2^Guangdong Key Laboratory of Food Intelligent Manufacturing, Foshan University, Foshan, China; ^3^Faculty of Veterinary and Agricultural Sciences, School of Agriculture and Food, University of Melbourne, Parkville, VIC, Australia

**Keywords:** Noni polysaccharides, fruit maturity stages, extraction yield, antioxidant activity, DNA protective effect

## Abstract

**Introduction:**

*Morinda citrifolia* L. (Noni) as an evergreen plant is a rich source of natural polysaccharides.

**Objective:**

The present work aims to investigate the maturation-related changes in polysaccharides of *Morinda citrifolia* L. (Noni) at five stages of maturity (stages from the lowest to highest degree – 1, 2, 3, 4, and 5).

**Methods:**

The chemical composition (carbohydrate, protein, uronic acid, and sulfate radical) of Noni polysaccharides was determined by different chemical assays. Ion chromatography system was used to analyze the monosaccharide composition, and the molecular weight was measured by HPGPC. The polysaccharides were also analyzed by FT-IR and their radical scavenging effect against DPPH, hydroxyl radicals and ABTS was evaluated. The UV-vis assay and gel electrophoresis assay were performed to investigate the DNA damage protective effect.

**Results:**

Results indicated the significant effect of fruit maturities on the extraction yields, molecular weights, uronic acid contents, sugar levels, monosaccharide compositions and proportions, antioxidant capacities, and DNA protective effects of Noni polysaccharides. However, no fruit maturity stage had prominent impact on the sulfuric radical contents and preliminary structure characteristics. Noni polysaccharides extracted at stage 5 (N5) had the largest extraction yield (8.26 ± 0.14%), the highest sugar content (61.94 ± 1.86%) and the most potent scavenging effect on DPPH (IC_50_: 1.06 mg/mL) and ABTS (IC_50_: 1.22 mg/mL) radicals. The stronger DPPH and ABTS radical scavenging activities of N5 might be contributed by its higher content of fucose and rhamnose and smaller molecular weight. Noni polysaccharides extracted at stage 4 (N4) showed the highest uronic acid content (4.10 ± 0.12%), and the superior performance in scavenging hydroxyl radicals and protecting DNA. The greater hydroxyl radical scavenging effect of N4 might be attributed to its higher percentage of the low molecular weight counterpart. Moreover, the DNA protective effects of N4 displayed a positive correlation with its hydroxyl radical scavenging ability.

**Conclusion:**

Overall, stage 4 and stage 5 could be ideal stages of fruit maturity aiming at high-quality Noni polysaccharides extraction. This study provided valuable information for the selection of suitable Noni polysaccharides to cater for various industrial applications.

## Introduction

*Morinda citrifolia* L. (Noni) is an evergreen plant native to tropical and subtropical regions (1). Noni fruit and its products have been attracting great attention from nutraceutical and pharmaceutical industries worldwide due to the increasing public awareness of their excellent pharmacological properties (2). Nutritional analysis of Noni has shown the presence of proteins, amino acids, organic acids, polyphenols, and vitamins in the fruit (3). In addition, Noni fruit is also a good source of natural polysaccharides (4). In Polynesia, India, Malaysia, Indonesia, and China, Noni fruit has been perceived as a traditional herb for over 2,000 years (5). Several *in vitro* and *in vivo* studies have reported that Noni fruit and its derived products exhibit anti-inflammatory, antidiabetic and antiproliferative activities as well as free radical scavenging activity (6, 7). As natural metabolites of Noni fruit, the Noni polysaccharides are claimed to be closely related to its therapeutic properties (8).

The changes of compositions in Noni fruit are induced by various biochemical and physiological reactions arising in fruit growth, development, and maturity (9), thereinto, the maturity of Noni fruit is vital to the quality of Noni polysaccharides. As the maturity progresses, plant polysaccharides are susceptible to diverse structure changes (debranching, acetylation/deacetylation, methylation/demethylation, depolymerization) catalyzed by enzymes. These modifications are correlated to softening and senescence of the plant, which can be observed along with the modifications in physiochemical properties (color, flesh breakdown, softening) and biological activities (antioxidant activity, anti-diabetic activity, immunomodulatory activity) since the early stage of ripening (10). The quality parameters of polysaccharides are highly important for selecting raw materials when producing high value-added Noni polysaccharides and their products. To find the ideal fruit maturity stage for extracting high-quality Noni polysaccharides, it is worthwhile to explore the differences of polysaccharides in Noni fruit during the ripening process. However, limited studies have been reported on this topic. Therefore, the present work intends to comprehensively investigate the variations in the extraction yields, physicochemical properties and bioactivities of Noni polysaccharides during fruit maturation. This information will provide practical insights into the selection of raw materials for extracting high-quality Noni polysaccharides.

## Materials and methods

### Material and chemicals

Noni fruits were purchased from Xisha Noni Co., Ltd. (Hainan, China). The pre-treatment process of Noni fruit is the same as in our previous study (11). The polysaccharides extracted from Noni fruit at different maturity stages (stage 1–stage 5) were coded N1, N2, N3, N4, and N5, respectively.

Bovine serum albumin (BSA), coomassie blue staining solution, 2,2-diphenyl-1-picrylhydrazyl (DPPH), and 2,2′-azino-bis (3-ethylbenzothiazoline-6-sulfonic acid) (ABTS) were obtained from Shanghai Aladdin Biochemical Technology Co., Ltd. (Shanghai, China). Pullulan standards of different molecular weights (4.4, 9.9, 21.4, 43.5, 124, 277, and 404 kDa) were acquired from Polymer Standards Service Co., Ltd. (Mainz, Germany). Standards of monosaccharides including fucose (Fuc), D-glucose (Glu), rhamnose (Rha), xylose (Xyl), mannose (Man), galactose (Gal), arabinose (Ara), glucuronic acid (GlcA), and galacturonic acid (GalA) and ctDNA were purchased from Sigma Chemical Co., Ltd. (St. Louis, MO, USA). pUC18 DNA was purchased from Solarbio Co., Ltd. (Beijing, China). All chemicals were of analytical reagent grade.

### Extraction and isolation of polysaccharides

In our previous study, hot-water extraction has been optimized by the response surface method (11). Briefly, Noni powder and ultrapure water were mixed at a ratio of 1–41.9 and heated at 77.7°C for 117.6 min, followed by the centrifugation at 3663 *g*, for 15 min. The supernatant was collected as the polysaccharide solution and then concentrated. The concentrate was then mixed with 4 times the volume of ethanol (100%) and placed in a refrigerator at 4°C for 12 h for precipitation. Afterward, the crude polysaccharides were harvested *via* centrifugation (3663 *g*, 15 min) and freeze-drying successively. The polysaccharide yield was calculated as follows:


$$\text{Yield (\%)}=\frac{\text{Weight of crude Noni polysaccharides (g)}}{\text{Weight of Noni fruit powders (g)}}$$

The purification of crude Noni polysaccharides followed the Sevag method, where the deproteinated solution was dialyzed and reprecipitated with ethanol, then collected and freeze-dried to give the purified polysaccharide for further analysis. The polysaccharides extracted from Noni fruit at different maturity stages (stage 1–stage 5) were coded N1, N2, N3, N4, and N5, respectively.

### Determination of chemical composition

The total carbohydrate content of Noni polysaccharides was measured by the phenol-sulfuric acid method (12). The protein content of Noni polysaccharide was determined following the Bradford method using BSA as the standard (13). The contents of uronic acids and sulfate radicals were determined by the corresponding reporter method (11).

### Determination of monosaccharide composition

The monosaccharide composition was evaluated as illustrated by Chen et al. (14). The polysaccharide samples were mixed with trifluoroacetic acid (2:1, w/v) and hydrolyzed at 105°C for 6 h. Subsequently, trifluoroacetic acid (4 M) was removed by rotary evaporation, then added with 5 ml methanol and further evaporated to remove residual trifluoroacetic acid (repeat 4 times). The final sample residue was dissolved in ultrapure water and the pH was adjusted to neutral for analysis. Ion chromatography system (ICS 5000, Dionex, CA) and Carbopac PA1 column (250 mm × 4 mm) were used to analyze the monosaccharide composition of the samples.

### Determination of molecular weights

High performance gel permeation chromatograph (HPGPC) with a triple column system was used to assess the molecular weight. ACQUITY APC AQ 900 2.5 μm column (4.6 mm × 150 mm), ACQUITY APC AQ 450 2.5 μm column (4.6 mm × 150 mm), and ACQUITY APC AQ 125 2.5 μm column (4.6 mm × 150 mm) were employed for the analysis of the molecular weight distribution. Both HPGPC and columns were purchased from Waters Corporation (USA). The analysis was performed with following settings: refractive index model, flow rate 0.4 ml/min, injection volume 40μL, mobile phase 100 mM NaNO_3_.

### Analysis of fourier transform-infrared (FT-IR) spectroscopy

In FT-IR analysis, potassium bromide (KBr) pellet was prepared by mixing 2 mg polysaccharide sample with KBr powder and further pressing. A vector 33 IR spectrophotometer (Bruker, Ettlingen, Germany) was used to measure the spectrum of the samples from KBr pellet in the range of 400–4000 cm^–1^.

### Analysis of antioxidant activity

DPPH and hydroxyl radicals scavenging activities were measured based on the previously reported methods (15, 16). The radical scavenging effect on ABTS was evaluated using the method of Xu et al. (17). Noni polysaccharides solution at different concentrations (0.2, 0.4, 0.6, 0.8, and 1.0 mg/ml) were selected for the analysis.

### Analysis of DNA damage protective effect

#### UV-*vis* assay

The protective effect against the DNA damage induced by hydroxyl radical was measured according to a previously reported method with some modifications (18). In brief, Noni polysaccharide phosphate buffer solution (0.2 M, pH 7.4) at different concentrations (2.0, 4.0, 6.0, 8.0, and 10.0 mg/ml), 0.5 mM Na_2_EDTA, 3.2 mM FeCl_3_, 5 mM H_2_O_2_, and 2.94 mM ctDNA were mixed in a ratio of 16: 4: 2: 3: 4 (v/v/v/v/v). To initiate the reaction, 0.075 ml 12 mM ascorbic acid was added in the mixture, then incubated in water bath at 55°C for 20 min. The reaction was terminated by adding 0.25 ml 0.6 M trichloroacetic acid, followed by the addition of 0.15 ml 0.35 M 2-thiobarbituric acid and heating at 105°C for 15 min. The mixture was determined at 530 nm and the buffer was used as the blank. The protection effect against DNA damage was calculated as follows:


$$\text{Protective effect (\%)}=\frac{A_{0}-(A_{1}-A_{2})}{A_{0}}\times 100$$

Where *A*_0_ is the absorbance of the control without Noni polysaccharides, *A*_1_ is the absorbance of the reaction system with Noni polysaccharides, *A*_2_ is the absorbance of the reaction system without ctDNA.

#### Gel electrophoresis assay

The evaluation of protective action for supercoiled pUC18 plasmid DNA against hydroxyl radical was according to the method of Wang et al. (18) with slight modifications. The reaction mixture contained 0.05 M PBS (9 μL), plasmid DNA (3 μL), Noni polysaccharide (8 mg/ml, 10 μL), 1 mM FeSO_4_ (4 μL), and 1 mM H_2_O_2_ (4 μL). The mixture was incubated at 37°C for 30 min, before mixing with loading buffer (0.05% bromophenol blue, 40 mM EDTA and 50% glycerol) at a ratio of 3:1 (v/v). Then, 6 μL of mixture was electrophoresed on 1% agarose gel for 30 min under 150 V condition. A Gel Documentation system (Hercules, CA, USA) was used to visualize and photograph the DNA gel.

### Statistical analysis

Each experiment was performed in triplicates, and the means ± standard deviations were reported as the results. The significant difference of mean values was measured by one-way ANOVA with Duncan’s multiple range tests at 95% confidence level using SPSS (SPSS, Chicago, USA). Pearson’s correlation analysis was used to identify the relationships between physicochemical properties and bioactivities of the samples. The Probit model from SPSS was applied to calculate the IC_50_ value.

## Results and discussion

### Appearance changes during ripening process

**FIGURE 1:**
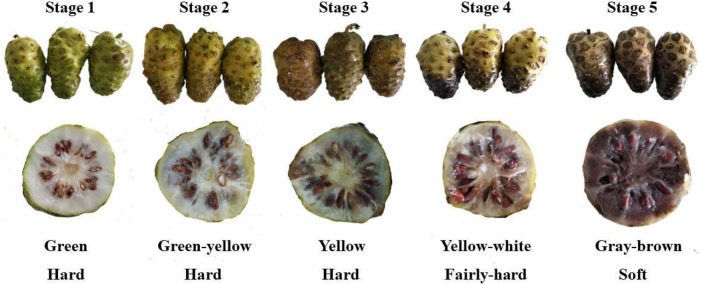
Noni fruit at different maturity stages (stages 1–5).

The appearance of Noni fruit at five maturity stages are shown in Figure 1. During fruit maturation, the color of fruit skin changed from green to brown. Carotenoids and chlorophyll (Chl) pigments have been found to be associated with color changes from green to yellow and brown in fruits. The degradation/catabolism of Chl is an enzymatic process with two pathways. The phytol and Mg are removed from Chl by the effects of chlorophyllase and Mg-dechelatase results in the opening of the backbone of the Chl ring structure subsequently leading to the deepening of peel color (19). On the other hand, Chl-degrading peroxidase can directly catabolize Chl pigment (20). The firmness of fruit remained hard in the early three stages but changed from hard to soft after stage 4. A decline in fruit firmness was associated with pectolytic enzymes, which are responsible for the polysaccharide degradation in the cell wall (21). The changes in the cell wall structure indicated the possible variation in the physicochemical properties of Noni polysaccharides during fruit softening.

### Chemical composition

Table 1 summarized the extraction yields and chemical compositions of Noni polysaccharide samples. The polysaccharide yield was increased in the following order: N5 > N4 > N3 > N2 > N1. N5 had the highest yield (8.26 ± 0.14%), while the lowest yield (3.86 ± 0.21%) was recorded for N1. Our result also revealed the similar sugar contents (61–62%) of N3, N4, and N5, which were significantly (*p* < 0.05) higher as compared to N1 and N2. During fruit ripening, cell wall polysaccharides such as pectin, cellulose and hemicellulose undergo solubilization, catabolism, and de-esterification by pectin methyl esterase, polygalacturonase, cellulase, and β-glucosidase, resulting in fruit softening as well as reduction in the molecular weight of cell wall polysaccharides. These results made the extraction of polysaccharides in stages 4 and 5 easier, with higher extraction rates and sugar contents in N4 and N5 than in the other three samples (22). The similar trend was also observed in the content of sulfuric radical. No protein was detected in all five Noni polysaccharide samples due to the complete removal of all free protein *via* pre-treatment. There were significant differences in the uronic acid level among all five sample, where N4 ranked the highest value. It is stated that the uronic acid concentration is positively correlated with the biological activities of polysaccharides (23), hence the N4 sample may have better bioactivities. Overall, stage 4 and stage 5 could be ideal maturity stages for the extraction of high-quality Noni polysaccharides.

**TABLE 1:** Chemical composition and extraction yield of Noni polysaccharide at different maturity stages.

Maturity	Extraction yield (%)	Sugar (%)	Sulfuric radical (%)	Protein (%)	Uronic acid (%)
N1	3.86 ± 0.21^e^	57.85 ± 1.25^c^	1.87 ± 0.15^c^	nd	1.19 ± 0.03^e^
N2	5.20 ± 0.18^d^	59.01 ± 0.98^b^	2.01 ± 0.16^b^	nd	2.62 ± 0.15^d^
N3	6.11 ± 0.09^c^	61.28 ± 1.80^a^	2.08 ± 0.20^a^	nd	3.56 ± 0.06^c^
N4	7.13 ± 0.12^b^	61.32 ± 2.01^a^	2.07 ± 0.18^a^	nd	4.10 ± 0.12^a^
N5	8.26 ± 0.14^a^	61.94 ± 1.86^a^	2.08 ± 0.17^a^	nd	3.88 ± 0.08^b^

The data are presented as mean values ± standard deviation. nd, not detected. Data marked with different letters are significantly different at *p* < 0.05.

### Molecular weight

Significant variations were observed among the molecular weight (M*_*w*_*) distribution for the Noni polysaccharide samples from five maturity stages. Table 2 showed the similar molecular weight values of N1, N2, N3, N4, and N5. Three polysaccharide fractions were detected in HPGPC, where the molecular weights were 97.96–129.50 kDa for peak A, 10.57–23.14 kDa for peak B, and 3.61–3.98 kDa for peak C. The main differences in molecular weight distribution occurred in the proportions of medium-M*_*w*_* (Peak B) and low-M*_*w*_* (Peak C) fractions. In N1, N2, and N3, the medium-M*_*w*_* fractions accounted for the main components, while the major constituents of N4 and N5 were low-M*_*w*_* fractions. The highest percentage of low-M*_*w*_* fraction (48.87%) was recorded for N5, while N2 contained the highest medium-M*_*w*_* fraction proportion (47.22%). Indeed, during fruit ripening, the polysaccharides in cell wall can be gradually degraded by pectinase, resulting in an increase of low-M*_*w*_* polysaccharide fractions and fruit softening (10). The results illustrated that the molecular weight distribution of Noni polysaccharides can be affected by the ripeness of Noni fruit.

**TABLE 2:** Retention time, molecular weight (M*_w_*), and amount of soluble polysaccharide fractions from five Noni polysaccharide samples.

Samples	Peak	Retention time (min)	M*_w_* (kDa)	Area account (%)
N1	Peak A	10.707	97.96	22.74
	Peak B	12.947	10.57	46.07
	Peak C	14.542	3.61	31.18
N2	Peak A	10.621	107.38	22.52
	Peak B	12.933	10.69	47.22
	Peak C	14.488	3.98	30.25
N3	Peak A	10.797	118.55	21.89
	Peak B	12.051	21.75	39.16
	Peak C	14.480	3.68	38.95
N4	Peak A	10.758	102.44	21.57
	Peak B	12.086	21.14	34.78
	Peak C	14.488	3.74	43.65
N5	Peak A	11.120	129.50	21.35
	Peak B	11.974	23.14	29.78
	Peak C	14.507	3.78	48.87

### FTIR analysis

**FIGURE 2:**
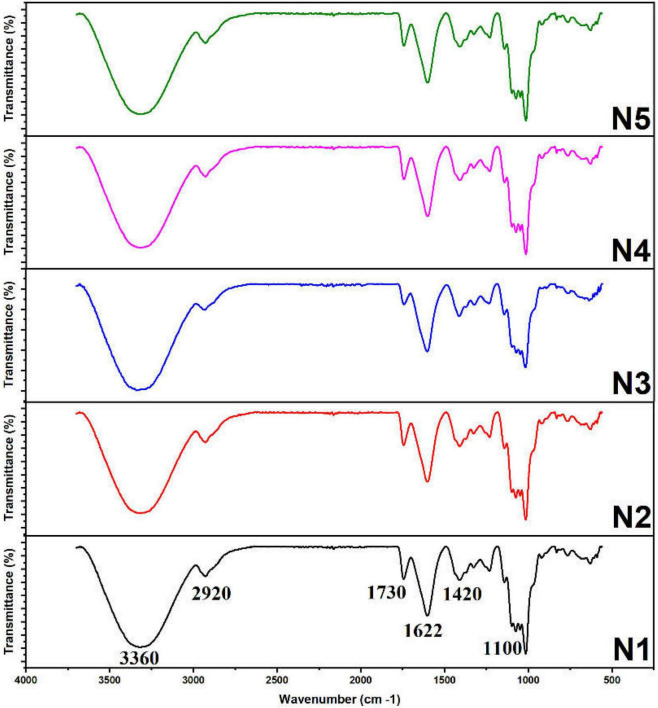
FT-IR spectra of five Noni polysaccharide samples extracted at different maturity stages.

As depicted in Figure 2, the FT-IR spectra of five Noni polysaccharide samples were recorded in the range of 4000–400 cm^–1^. All samples showed characteristic polysaccharide signals at around 3360, 2920, 1730, 1622, 1420, and 1100 cm^–1^. The strong absorption signals at around 3360 and 1622 cm^–1^ were ascribed to the deformation vibration and stretching vibration of hydroxyl clusters (12). The absorption bands at around 2920 cm^–1^ were characteristic of the axial stretching of the C-H bond of methyl groups. Absorption signals at around 1730 and 1622 cm^–1^ corresponded to C=O stretching vibration of carboxylic acid, confirming the presence of certain amount of uronic acid in polysaccharide samples (24). The existence of carbonyl clusters was also revealed by the band at 1420 cm^–1^. In addition, prominent signals in the range of 1000–1200 cm^–1^ implied that all samples contained pyranose ring structure and glycosidic bond. The FT-IR spectra of the five samples were similar, which indicated that the maturity of Noni fruit had no significant impact on the type of glycosidic bonds and the primary structure of Noni polysaccharides.

### Monosaccharide composition

The monosaccharide concentrations of Noni polysaccharide samples are listed in Table 3. Results demonstrated that the monosaccharide constituents of N2, N3, N4, and N5 were measured as Fuc, Ara, Gal, Glu, Xyl, Man, Rha, GalA, GluA, which are in good agreement with the previous report (11). However, Xyl and Man were not detected in N1. The maximum concentrations of Gal (3.909 ± 0.104 mg/L) and Man (0.420 ± 0.011 mg/L) were found in N3, while the highest values of Ara (3.025 ± 0.015 mg/L) and GalA (3.875 ± 0.115 mg/L) were observed in N4. Moreover, the N5 sample had significantly higher levels of Fuc (0.585 ± 0.023 mg/L) and Glu (3.674 ± 0.029 mg/L) than the other samples. Similar trends in glucose content were reported in sweet cherries and Natal plum (25, 26), where the glucose concentrations also peaked in the final maturity stage. As ripening progresses, glucose increase due to the action of the enzyme invertase through glycolysis in fruits and vegetables (27). The dominant monosaccharides in N3, N4, and N5 were Ara, Gal, Glu and GalA, while N1 was mainly made up of Fuc, Rha, GalA, and GluA. The composition of N2 sample was closer to N3, N4, and N5, which was composed of Ara, Gal, and GalA. These results demonstrated that the content and constituent of monosaccharides in Noni polysaccharide samples were influenced by the fruit maturity stages.

**TABLE 3:** Monosaccharide concentrations (mg/L) of Noni polysaccharide samples extracted at five stages of maturity.

Maturity	Fuc	Ara	Gal	Glu	Xyl	Man	Rha	GalA	GluA
N1	0.400 ± 0.020	0.088 ± 0.009	0.070 ± 0.012	0.010 ± 0.002	nd	nd	0.180 ± 0.005	0.967 ± 0.021	0.222 ± 0.011
N2	0.410 ± 0.017	1.396 ± 0.012	2.905 ± 0.030	0.010 ± 0.003	0.037 ± 0.009	0.315 ± 0.012	0.190 ± 0.003	2.424 ± 0.144	0.195 ± 0.006
N3	0.422 ± 0.011	2.807 ± 0.012	3.909 ± 0.104	2.992 ± 0.042	0.078 ± 0.005	0.420 ± 0.011	0.192 ± 0.002	3.319 ± 0.044	0.240 ± 0.016
N4	0.536 ± 0.021	3.025 ± 0.015	3.282 ± 0.066	2.895 ± 0.036	0.078 ± 0.006	0.339 ± 0.006	0.201 ± 0.002	3.875 ± 0.115	0.222 ± 0.005
N5	0.585 ± 0.023	2.816 ± 0.021	3.799 ± 0.031	3.674 ± 0.029	0.015 ± 0.002	0.302 ± 0.002	0.198 ± 0.005	3.668 ± 0.075	0.207 ± 0.005

nd, not detected.

### Antioxidant capacity

#### Scavenging activity on DPPH radical

**FIGURE 3:**
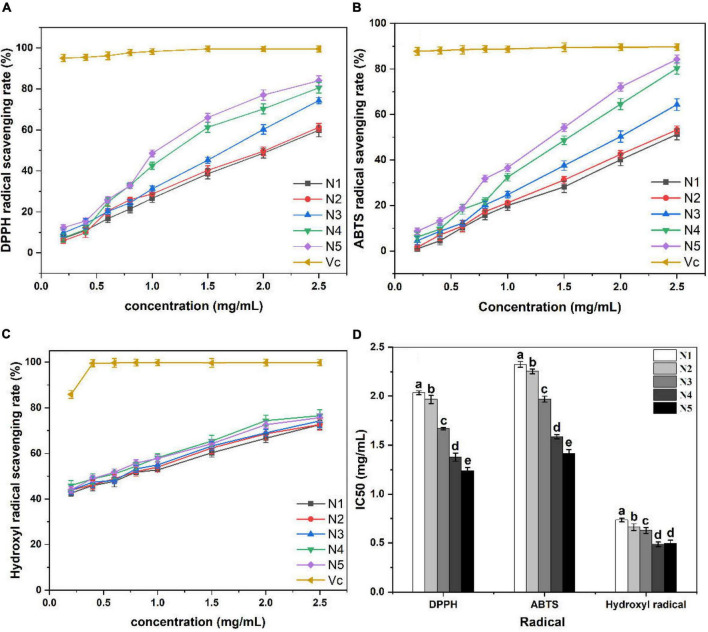
Scavenging activity of Noni polysaccharide samples on DPPH **(A,D)**, ABTS **(B,D)**, and hydroxyl radical **(C,D)**. Data marked with different letters are significantly different at *p* < 0.05.

As shown in Figures 3A, D, all polysaccharide samples demonstrated DPPH radical scavenging activities that were positively correlated with the tested concentrations. However, the ascorbic acid group showed the significantly (*p* < 0.05) higher DPPH radical scavenging capacity than the five polysaccharide samples. In fact, the IC_50_ values for scavenging of DPPH radicals were measured to be 2.03 mg/ml (N1), 1.96 mg/ml (N2), 1.66 mg/ml (N3), 1.37 mg/ml (N4), and 1.23 mg/ml (N5). Therefore, it can be concluded that the N5 sample exhibited the superior DPPH radical scavenging ability among the five samples tested. Antioxidants can scavenge DPPH radicals by donating hydrogen to form stable DPPH-H molecules with DPPH radicals (28). It was evident that N5 showed the strongest proton donating ability and could serve as DPPH radical scavengers, acting possibly as antioxidants in the five polysaccharide samples.

#### Scavenging activity on ABTS radical

The results of the ABTS radical scavenging ability of the five samples are depicted in Figures 3B, D. All samples performed the ABTS scavenging activity in a dose-response manner. N5 exhibited higher ABTS radical scavenging ability as compared to the other polysaccharide samples at each concentration, albeit being significantly (*p* < 0.05) lower than that of the ascorbic acid group. The blue–green ABTS^+^ free radicals will be reduced to colorless ABTS under the action of antioxidants (29). The lowest IC_50_ value was observed in the N5 sample (1.41 mg/ml), which validated that stage 5 could be the optimum maturity stage for extracting Noni polysaccharides with high reducing power to scavenge ABTS radicals.

#### Scavenging activity on hydroxyl radical

Hydroxyl radicals are the most reactive radicals, which can cause oxidative damage to human tissue and induce severe cell death (30). It is important to find natural antioxidants to scavenge hydroxyl radicals since there are no enzymatic systems known to neutralize them in the human body. The results of the hydroxyl radical scavenging test are presented in Figures 3C, D, which revealed that all measured polysaccharide samples exerted dose-dependent scavenging effects on hydroxyl radicals. N4 exhibited stronger hydroxyl radical scavenging ability than the other four samples at the concentrations ranging from 0.2 to 2.5 mg/ml. Moreover, the IC_50_ values of all tested samples were different, which followed the order of N4 < N5 < N3 < N1 < N2. Generally, polysaccharides with high uronic acid content and low molecular weight possess strong antioxidant activity (27). In this part, the IC_50_ values of N5 (0.49 mg/ml) was not significantly different from N4 (0.48 mg/ml) with higher uronic acid content (*p* > 0.05), which may be related to its smaller molecular weight.

### DNA protective effect

#### UV-*vis* assay

**FIGURE 4:**
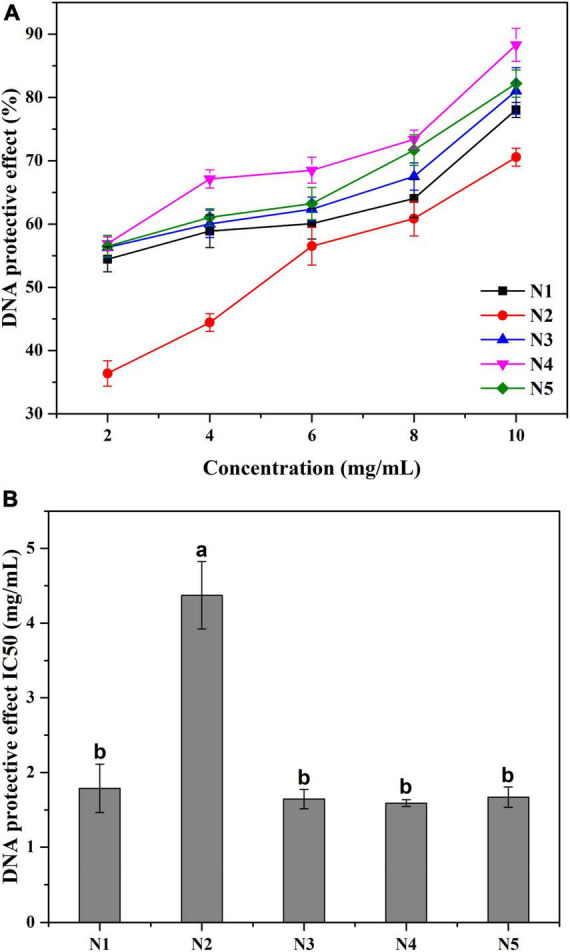
**(A)** The protective effect against ^•^OH radical induced DNA damage at different concentrations of Noni polysaccharide samples. **(B)** The IC_50_ value of different Noni polysaccharide samples. Data marked with different letters are significantly different at *p* < 0.05.

Free radicals especially hydroxyl radicals, may cause a variety of DNA damages to the human body, such as altering signaling cascades, making gene misexpression and increasing replication errors. As shown in Figure 4, all five samples showed prominent DNA protective abilities in a concentration-dependent manner. At the tested concentrations of 2–10 mg/L, the strongest DNA protective action was detected in N4 at 10 mg/ml with the protective rate as 88.31%. Meanwhile, the IC_50_ values of the five samples showed a similar trend to the hydroxyl radical scavenging test. N4 had the lowest IC_50_ value of the DNA protective effect (1.59 mg/ml), while the IC_50_ value of N2 was significantly (*p* < 0.05) higher when comparing with the other samples. In this experiment, DNA damaged by hydroxyl radicals can easily form pink acidic thiobarbituric acid-reactive substance (TBARS) under acidic and high-temperature conditions (31). The results suggested that all Noni polysaccharide samples could suppress the generation of TBARS and thus protect DNA against hydroxyl radical-induced damage. It is worth noting that the DNA protective effects of five samples were positively related to their hydroxyl radical scavenging abilities.

#### Gel electrophoresis assay

Supercoiled pUC18 plasmid DNA has been extensively employed as a test model for detecting the potential active substances that protect DNA against oxidative damage (32). For the pUC18 plasmid DNA model, three forms can be displayed on gel electrophoresis, including Form 1 (supercoiled form), Form 2 (open circular form) and Form 3 (linear form). The migration speed of three forms followed the order of Form 1 > Form 3 > Form 2. Generally, undamaged plasmid DNA mainly consists of the supercoiled form, while the open circular form and linear form will be generated once the DNA is damaged.

**FIGURE 5:**
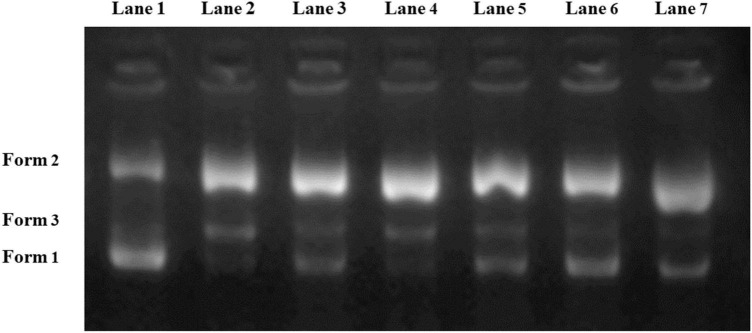
Oxidative damage protective ability of Noni polysaccharide to pUC18 supercoiled plasmid DNA. Lane 1: native DNA; Lane 2: DNA + H_2_O_2_ + FeSO_4_; Lane 3–Lane 7: DNA + Noni polysaccharide (N1, N2, N3, N4, N5) + H_2_O_2_ + FeSO_4_.

As illustrated in Figure 5, the control group (Lane 1) mainly displayed in the supercoiled band (Form 1). In the meantime, Lane 2 showed that most of the normal DNA with supercoiled structure was converted into linear form and open circular form after adding H_2_O_2_ and FeSO_4_. Lane 3–Lane 7 exhibited the protective effects of the five samples on DNA. According to the electrophoresis results, N4 showed the strongest DNA protective ability since its Form 1 band was brighter than that of the other samples. The electrophoresis feature of N2 (Lane 4) was similar to that of Lane 2, indicating that N2 had the lowest DNA protective activity. Although the reaction system was different from the UV-*vis* method, these results further validated N4 as the most promising protectant against DNA strand scission caused by hydroxyl radicals.

### Correlation analysis

Pearson analysis was performed to elucidate the correlation between the physicochemical characteristics and biological activities of the five Noni polysaccharide samples. As shown in Table 4, it can be seen that the antioxidant ability of each polysaccharide sample showed a prominent linear correlation with its sugar content, monosaccharide concentration and the ratio of different molecular weight fractions. The contents of sugar, Fuc and Rha revealed a strong negative correlation with the antioxidant abilities of Noni polysaccharides (*p* < 0.05), indicating that the Noni polysaccharide with high sugar, Fuc and Rha contents had superior antioxidant abilities. It was reported that the Fuc and Rha exerted antioxidative properties by scavenging reactive oxygen species (33), which is consistent with our correlation analysis. Furthermore, the ratio of three molecular weight fractions (high-M*_*w*_*, medium-M*_*w*_*, and low-M*_*w*_*) were significantly correlated with the IC_50_ value of radicals scavenging activity (*p* < 0.05). For the IC_50_ value of radical assay, the ratio of high-M*_*w*_* and medium-M*_*w*_* component showed a positive correlation (*p* < 0.05), while the ratio of the low-M*_*w*_* component demonstrated a negative correlation (*p* < 0.05), indicating that the Noni polysaccharide with a smaller molecular weight showed better radical scavenging ability. Previous studies also reported the superior antioxidative ability of low-molecular-weight polysaccharides, which was attributed to their larger surface area and greater water solubility facilitating the reaction with radicals (34, 35). From Table 4, the contents of uronic acid and GalA also displayed a significant linear correlation with the IC_50_ value of hydroxyl assay with the coefficient of –0.991 and –0.913 (*p* < 0.05). The results showed that polysaccharides with high uronic acid content had stronger hydroxyl radical scavenging ability, which was similar to other polysaccharides reported before (36). Although the physicochemical properties of Noni polysaccharide did not show an obvious correlation with the DNA protective effect, a close positive correlation was seen between the DNA protective effect and radical scavenging activity (not shown in Table 4). Based on our analysis, the bioactivities of Noni polysaccharide samples were not impacted by a single factor but a function of multiple factors.

**TABLE 4:** Matrix for correlation analysis.

Value	IC_50_ of DPPH assay	IC_50_ of ABTS assay	IC_50_ of hydroxyl assay	IC_50_ of DNA protective effect
Sugar content (%)	-0.926*	-0.901*	-0.883*	-0.450
Sulfuric radical (%)	-0.778	-0.748	-0.803	-0.128
Uronic acid (%)	-0.878	-0.856	-0.991*	-0.266
Fuc (mg/L)	-0.950*	-0.968**	-0.936*	-0.432
Ara (mg/L)	-0.861	-0.832	-0.870	-0.333
Gal (mg/L)	-0.696	-0.664	-0.721	-0.011
Glu (mg/L)	-0.857	-0.806	-0.840	-0.642
Xyl (mg/L)	-0.313	0.264	-0.402	-0.123
Man (mg/L)	-0.527	-0.487	-0.587	0.085
Rha (mg/L)	-0.892*	-0.884*	-0.966**	-0.205
GalA (mg/L)	-0.878	-0.856	-0.913*	-0.255
GluA (mg/L)	-0.077	0.027	-0.060	-0.732
High-M*_*w*_* parts (%)	0.989**	0.977**	0.950*	0.515
Medium-M*_*w*_* parts (%)	0.987**	0.984**	0.909*	0.622
Low-M*_*w*_* parts (%)	-0.989**	-0.985**	-0.914*	-0.615

*p < 0.05.

**p < 0.01.

## Conclusion

Remarkable changes in physicochemical properties and bioactivities of Noni polysaccharides occurred during the fruit ripening process. The highest polysaccharide yield and sugar content were seen at stage 5, whereas the highest uronic acid level was recorded at stage 4. Our results confirmed that the monosaccharide concentrations and proportions, molecular weights, antioxidant activities and DNA protective abilities of Noni polysaccharides varied significantly among five maturity stages, which indicates the necessity for selecting suitable polysaccharides to meet different industrial and nutritional requirements. However, the maturity stages have no remarkable influence on the sulfuric radical content and preliminary structure. Noni polysaccharide extracted at stage 5 showed the strongest scavenging activities against DPPH and ABTS radicals, while the hydroxyl radical scavenging capacity reached the peak at stage 4. Overall, stage 4 and stage 5 are the ideal stages of maturity for extracting high-quality Noni polysaccharides.

## Data availability statement

The original contributions presented in this study are included in the article/supplementary material, further inquiries can be directed to the corresponding authors.

## Author contributions

JC: conceptualization, experiment, data analyses, and writing. ZL: writing. JL: experiment and data analyses. HL and ZH: review and editing. MM: experiment and editing. XZ: supervision, funding, and review. All authors contributed to the article and approved the submitted version.
